# Ion and lipid signaling in apical growth—a dynamic machinery responding to extracellular cues

**DOI:** 10.3389/fpls.2015.00816

**Published:** 2015-10-06

**Authors:** Rui Malhó, Susana Serrazina, Laura Saavedra, Fernando V. Dias, Reiaz Ul-Rehman

**Affiliations:** BioISI – Biosystems & Integrative Sciences Institute, Faculdade de Ciências, Universidade de Lisboa, Lisboa, Portugal

**Keywords:** Ca^2+^, cyclic nucleotides, syntaxins, phosphoinositides, signaling, tip growth

## Abstract

Apical cell growth seems to have independently evolved throughout the major lineages of life. To a certain extent, so does our body of knowledge on the mechanisms regulating this morphogenetic process. Studies on pollen tubes, root hairs, rhizoids, fungal hyphae, even nerve cells, have highlighted tissue and cell specificities but also common regulatory characteristics (e.g., ions, proteins, phospholipids) that our focused research sometimes failed to grasp. The working hypothesis to test how apical cell growth is established and maintained have thus been shaped by the model organism under study and the type of methods used to study them. The current picture is one of a dynamic and adaptative process, based on a spatial segregation of components that network to achieve growth and respond to environmental (extracellular) cues. Here, we explore some examples of our live imaging research, namely on cyclic nucleotide gated ion channels, lipid kinases and syntaxins involved in exocytosis. We discuss how their spatial distribution, activity and concentration suggest that the players regulating apical cell growth may display more mobility than previously thought. Furthermore, we speculate on the implications of such perspective in our understanding of the mechanisms regulating apical cell growth and their responses to extracellular cues.

## Introduction

Apical tip growth is a form of cell extension common in all eukaryotes from rhizoids, pollen tubes, fungal hyphae to nerve cells. This growth form serves as a paradigm for cell polarity because cell extension is restricted to a narrow zone at the apex (Cheung and Wu, 2008). These cells are recognizably excellent models for cell research, particularly suitable for investigations on polarization, signal transduction, channel and ion flux activity, gene expression, cytoskeleton and wall structure, membrane dynamics and even cell-cell communication (Malhó et al., 2000; Onelli and Moscatelli, 2013; Sekereš et al., 2015).

**FIGURE 1:**
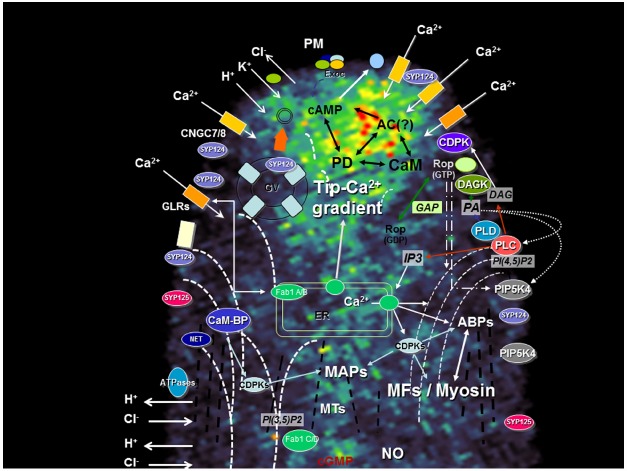
**Apical region of a growing pollen tube depicting the main signaling transduction pathways and their components.** A network between the different signaling pathways foresees the existence of a highly dynamic mechanism capable to interpret simultaneous extracellular cues and maintain polarity. The diagram is superimposed on a confocal image of a growing tobacco pollen tube loaded with FM1-43, a probe for endo-exocytosis [for methods see Camacho and Malhó (2003)]. ABPs, actin-binding proteins; AC, Adenylyl cyclase; CaM-BP, Calmodulin-binding protein; CDPK, Ca^2+^ dependent protein kinase; CNGC, cyclic nucleotide gated channel; DAG, diacylglycerol; DAGK, DAG kinase; Exoc, Exocyst; Fab1, PIKfyve/Fab1 kinase; GAP, Rop GTPase activating protein; GLR, Glutamate-like receptor; GV, Golgi vesicle; IP3, Inositol 1,4,5 triphosphate; MAPs, microtubule-associated protein; MFs, microfilaments (dashed white bars); MTs, microtubules (dashed black bars); NET, plant-specific Networked protein; NO, nitric oxide; PA, phosphatidic acid; PD, Phosphodiesterase; PI(3,5)P2, phosphatidylinositol-(3,5)-bis phosphate; PI(4,5)P2, phosphatidylinositol-(4,5)-bis phosphate; PIPK, phosphatidylinositol kinase; PLC, phospholipase C; PLD, phospholipase D; PM, plasma membrane; PMEs, pectin-methyl-esterases; R, IP3 receptor; SYP, syntaxin. Barbed arrows (↗) indicate direction of flux; Triangle arrows (△) indicate potential cross-regulatory effects. The curved MFs lines represent the actin fringe with larger cables extending to the sub-apex (and connecting to the plasma membrane) but not to the apical zone. The microtubules, where a fringe is not so visible, are represented as straight lines.

As in any topic in science, advances in knowledge are dictated by current state of technology, the models under study and pre-existing ideas (typically represented in static diagrams such as the one represented in Figure 1). In the case of apical growth in plants, the outstanding technological advance registered in the past two decades, caused a perspective change from a morphological/structural approach to a dynamic/functional approach. When cell biology tools were predominantly used, researchers focused on larger cells that would grow straight and fast under *in vitro* conditions and would tolerate more harsh experimental conditions (e.g., microinjection, synthetic dyes, use of antibodies, and fixation methods). *Lilium longiflorum* was the paradigmatic example and underlying most experimental design was the hypothesis that if a molecule was important for tip growth, then its concentration should be higher in the apex (Malhó et al., 2000). The development of molecular tools in parallel with non-invasive methods to study live cells, made *Arabidopsis thaliana* a more accessible model and diversified tip growth studies. With the burst of genomics, a wider range of species (e.g., *Physcomitrella patens*) became also more prone to functional studies. Simple traits like *fast growth rate* or *straight growth direction* no longer hold as “universal” physiological controls and data interpretation must consider cellular and tissue variability.

The diversity of species and characteristics of cells under study naturally generated a diversity of results (e.g., the natural growing environment of an Arabidopsis pollen tube is different from one of lily, from a root hair or from a moss rhizoid). Distinct asymmetric localization of components of the apical growth machinery (summarized in the diagram of Figure 1) were reported as part of new studies or reassessment of previous data. This, in turn, generated discrepancies, challenged old ideas and opened new perspectives. The architecture of the actin cytoskeleton (Vidali et al., 2009) and the secretory activity (Zonia and Munnik, 2008) are just two examples that challenged the “all-in-the-tip” concept and highlighted the importance of dynamics at the sub-apical region (Cheung and Wu, 2008; Sekereš et al., 2015). Here we explore the example of four different types of proteins recently studied by our group and which were found to be important for tip growth. In all four cases, we found that changes in growth pattern (e.g., redirectioning of growth axis, transient loss of polarity and recovery, oscillatory growth rates) were accompanied by protein delocalization. We discuss the implications of such findings and outline hypothesis to interpret the mechanisms underlying this complex machinery and their responses to extracellular stimuli.

## Cyclic Nucleotide Gated Channels—Mobile and Flexible Ion Influx

**FIGURE 2:**
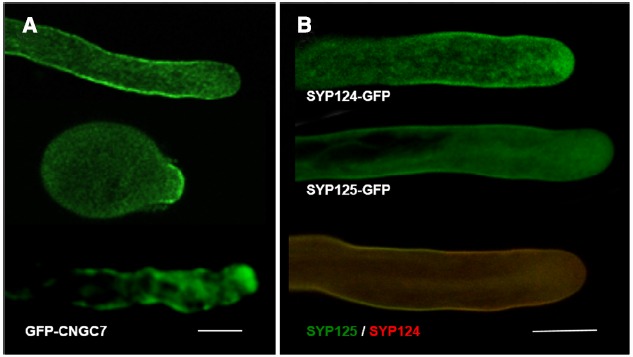
**Confocal Imaging of GFP constructs of CNGC7, SYP124 and SYP125. (A)** Arabidopsis pollen tubes expressing GFP-CNG7, in different growth phases—straight growth; upon germination; wiggling growth. For methods see Tunc-Ozdemir et al. (2013). Bar = 10 μm. **(B)** Tobacco pollen tubes expressing SYP124-GFP (upper image), SYP125-GFP (middle image), and co-expressing both constructs (lower image; SYP124 signal in red and SYP125 signal in green). For methods see Ul-Rehman et al. (2011). Bar = 10 μm.

Ca^2+^ signaling plays a key role in all aspects of plant development including apical growth. Namely, a tip-high gradient of cytosolic free ([Ca^2+^]_*c*_) resulting from influx of extracellular Ca^2+^ seems crucial to establish polarity (Hepler et al., 2012). Evidence from pharmacological and genetic approaches indicate this influx occurs through at least two different types of Ca^2+^ -permeable channels—glutamate receptor-like proteins (GLRs) and cyclic nucleotide gated channels (CNGCs; Michard et al., 2011; Tunc-Ozdemir et al., 2013). CNGCs are cation channels with varying degrees of ion conduction selectivity harboring a cyclic nucleotide-binding domain and a calmodulin binding domain (Zelman et al., 2012). They can therefore integrate signals from distinct transduction pathways and could be functioning in a way that indirectly triggers a Ca^2+^ release from an internal store (Spalding and Harper, 2011). CNGCs were shown to be essential in tip-growing cells (Frietsch et al., 2007; Tunc-Ozdemir et al., 2013) and, in straight growing pollen tubes, GFP-CNGC7 was found to preferentially localize to the plasma membrane at the flanks of the growing tip (Figure 2A, top image). But at the onset of germination, perhaps the phase where apical Ca^2+^ influx is more relevant to establish a growth axis, the GFP-CNG7 signal was higher at the apex (Figure 2A, middle image). Similar observations were made in pollen tubes recovering and/or reorienting the growth axis (Figure 2A, lower image) suggesting that cells regulate the fine tuning of protein localization in response to extracellular cues. FRAP experiments of GFP fused to RLK (Receptor-Like-Kinase; Lee et al., 2008) showing apical fluorescence recovery support such hypothesis.

Tip growing cells not only have to frequently adjust direction of growth axis but they also experience oscillations in growth rates. Several types of growth fluctuations (of asymmetric periodicity and intensity) have been observed and they seem to vary according to species and cell type. Whether these oscillations have any special physiological meaning or whether they are just a “built-in” characteristic is not clear. [Ca^2+^]_*c*_ exhibits changes that correlated to such fluctuations of growth rates and reorientation of growth axis (Castanho Coelho and Malhó, 2006) so it is highly plausible that other components of the polarity mechanism (e.g., lipids and membrane-associated proteins) also exhibit changes in activity and/or localization. The timing of our observations (and the inherent physiological status of the cells) may thus influence our reports and help to explain some apparent discrepancies that exist in the literature.

## PIP Kinases—Versatile and Key Transducers of “Signal to Form”

In the last years PtdIns(4,5)P_2_ and its synthesizing enzyme, phosphatidylinositol phosphate kinase (PIPK), have been intensively studied in plant cells, revealing a key role in the control of polar tip growth. Using fluorescence markers fused to the pleckstrin homology (PH) domain of the human PLCd1, PtdIns(4,5)P_2_ was found to accumulate at the tip of growing apical cells (Kost et al., 1999; Dowd et al., 2006; Helling et al., 2006; Ischebeck et al., 2008; Sousa et al., 2008). Analysis of the PIPK members from *Arabidopsis thaliana*, *Oryza sativa*, and *Physcomitrella patens* showed that they share some regulatory features with animal PIPKs but also exert plant-specific modes of regulation (Saavedra et al., 2012). Deletion or overexpression of these lipid kinases were found to cause distinct phenotypes and perturbations in cellular processes such as cell wall deposition, endocytosis, and actin bundling (Ischebeck et al., 2008, 2010; Kusano et al., 2008; Sousa et al., 2008; Stenzel et al., 2008; Zhao et al., 2010; Saavedra et al., 2011). Interestingly, in actively growing pollen tubes, all the six PIPK isoforms (*AtPIP5K10*, *AtPIP5K11*, *AtPIP5K2*, *AtPIP5K4*, *AtPIP5K5*, and *AtPIP5K6*) were found to localize preferentially at the flanks of the tube apex and not superimposed with the highest PtdIns(4,5)P_2_ concentration. However, it was also observed that the region displaying the highest protein fluorescent signal would change according to speed of growth and reorientation of the growth axis—slower growth resulting in delocalization from the flanks to the apex (e.g., Figure 6 of Ischebeck et al., 2008; Sousa et al., 2008). It has been suggested that distinct localization patterns of proteins may be the consequence of interactions with specific partners, which recruit them to different functional microdomains (Ischebeck et al., 2010). For example, PtdIns(4,5)P_2_ could be channeled to targets via specific interactions PIPK-downstream effectors (Heilmann and Heilmann, 2015) resulting in differential cellular responses and phenotypes as observed upon deletion of AtPIP5Ks (Ischebeck et al., 2008, 2010; Kusano et al., 2008; Sousa et al., 2008).

Phosphatidylinositol phosphate kinases have membrane occupation and recognition nexus (MORN) motifs which are thought to be the plasma membrane localizing module (Kusano et al., 2008) but other modules were shown to be important for correct subcellular localization (Mikami et al., 2010; Stenzel et al., 2012). E.g., AtPIP5K5 and NtPIP5K6-1 require non-conserved linker (LIM) domain for correct localization in pollen tubes, as the deletion of the N-terminal and MORN domain did not affect their apical plasma membrane localization (Stenzel et al., 2012). It is thus possible that protein modules responsible for plasma membrane localization are distinct in each PIPK allowing a fine tuning that depends on differences in physiological and/or developmental status of cells, such as polarized and non-polarized. Full comprehension of the localization mechanisms will probably involve comparison of the function of the MORN, LIM, and kinase domains of every PIPKs in cells exhibiting the same stage in development.

## Syntaxins—Spatial Targeting of Secretion and Membrane Recycling

Apical growth occurs by continuous vesicle secretion and delivery of new wall material. Therefore, the exact sub-cellular location of endocytic and exocytic domains is essential to determine cellular responses and reshape form. In pollen tubes, it was previously assumed that exocytosis events occur mostly at the extreme apex, where [Ca^2+^]_*c*_ is higher, while membrane recycling (endocytosis) would take place further back from the tip, at the flanks of the apex and/or at sub-apical regions (Picton and Steer, 1981; Castanho Coelho and Malhó, 2006). Recent data questioned this paradigm suggesting that the preferential location for fusion is on a limited membrane domain at the sub-apical flanks and not at the extreme apex (Bove et al., 2008; Zonia and Munnik, 2008). Similar observations have been made in root hairs (Ovecka et al., 2005). The hypothesis of two endocytic modes co-existing according to the growth conditions—a clathrin-dependent and an independent one—was raised (Camacho and Malhó, 2003; Monteiro et al., 2005) and experimental evidence to support it was obtained by Moscatelli et al. (2007) and McKenna et al. (2009). We have also obtained evidence that two pollen-specific SNAREs (for soluble N-ethylmaleimide sensitive factor attachment protein receptor), syntaxins SYP125 and SYP124 (Silva et al., 2010; Ul-Rehman et al., 2011) have a complementary, not fully overlapping, dynamic distribution (Figure 2B). Similarly to PIPK proteins, the observed changes in protein accumulation at the plasma membrane might reflect specific interactions with unidentified targets (e.g., PIP2 and phosphatidic acid) which, under natural growth conditions of pollen tubes, could translate into discrete asymmetric secretory events and relate to the two endocytic modes. Indirect support for such hypothesis derives from our observations that in pollen tubes, [Ca^2+^]_*c*_ and membrane fusion exhibit frequent non-linear changes correlated to growth rates and reorientation of growth axis (Castanho Coelho and Malhó, 2006). Over-expression of PIP5K4 or Rab GTPases was also found to perturb SYP124 localization (Silva et al., 2010). The changes in SYP124 and SYP125 distribution observed upon growth modulation are thus likely to reflect membrane dynamics and a repositioning of the vesicle’s docking machinery upon intra- and extracellular cues. In nerve cells, syntaxins were reported to be involved in rapid and slow endocytosis (Xu et al., 2013) and could thus act as regulators of both endo- and exocytosis. VAMP726, another member of the SNARES family, was also shown to mediate fusion of endo- and exocytic compartments in pollen tube tip growth (Guo and McCubbin, 2012). A similar role has just been proposed for the exocyst (Jose et al., 2015), a complex known to be present in tip growing cells (Zárský et al., 2013).

Similarly to our CNGC observations, these findings highlight the importance of syntaxins in secretion and tip growth but must be interpreted considering that the localization reported reflects protein accumulation and not necessarily activity.

## FAB Kinases- Dynamics in the Secretory Pathway

Most of the data now available relating apical polarity and growth focused on the role of plasma membrane (integral or associated) events. The plasma membrane and its links to the cell wall and the cytoskeleton are the obvious main targets for perceiving and transducing both intra- and extracellular cues. Notwithstanding, we have recently obtained data suggesting that PIKfyve/Fab1 kinases localized to the endomembrane compartment are also involved in the regulation of plasma membrane recycling events and thus in the maintenance of polarity (Serrazina et al., 2014).

In plants, the Fab1 phosphatidylinositol-3-monophosphate 5-kinases produce phosphatidylinositol (3,5)-bisphosphate [PtdIns(3,5)P_2_], a phosphoinositide implicated in endomembrane trafficking and pH control in the vacuole (Dove et al., 2009; Bak et al., 2013). In pollen tubes, we found that AtFAB1B-GFP had a highly mobile reticulate-like distribution, distinct from γ-TIP (Serrazina et al., 2014) and decorating the sub-apical region in a manner similar to the actin cytoskeleton—a sort of V-shaped collar that adjusts and “accompanies” the reorientation of the growth axis (Vidali et al., 2009). This suggests that the protein is localized in protein storage vacuoles, endoplasmic reticulum and possibly trans-Golgi network mediating transport to and from the plasma membrane (Gary et al., 1998; Wang et al., 2011). Additionally, we found that FAB1 deletion resulted in lower internalization rates and perturbed secretion and deposition of new wall material (Serrazina et al., 2014). Analogous observations were made in root hairs (Hirano et al., 2011). FAB1 deletion was also found to impair acidification of the endomembrane compartment and to cause abnormal pollen tube diameter (Serrazina et al., 2014). The perturbation of proton gradients across membranes was reported to affect membrane curvature and vesicularization (Hope et al., 1989), protein sorting (Hurtado-Lorenzo et al., 2006), and ion fluxes, all of which are processes essential for tip growth.

These results confirm that apical polarity is not solely dependent on a positive feedback mechanism based on a single protein or ion influx localization but rather on an orchestrated network of signals. Gui et al. (2014) have recently found that overexpression of LePRK1, a pollen-specific and plasma membrane-localized receptor-like kinase, dramatically affects tube morphology in a process that can be counterbalanced by an actin bundling protein (PLIM2a) in a Ca^2+^-responsive manner.

## Conclusion and Perspectives

The establishment and maintenance of apical polarity relies on a dynamic, mobile network of signaling mechanisms. Currently, our conceptual models focus mostly on apical and sub-apical localization (of ions, proteins, lipids), particularly at the plasma membrane (or associated with). Here we provided examples of four classes of proteins related to ion and lipid signaling which indicates that a careful analysis of localization, activity and mobility is required in order to fully assign their role in apical growth. This rationale can probably be extended to other equally relevant signaling components identified in these cells (e.g., Rop GTPases, Ca^2+^-dependent protein kinases, actin and actin-binding proteins, etc; Figure 1; for a review see Onelli and Moscatelli, 2013). The results obtained with FAB kinases further suggest the importance of plasma membrane—endomembrane signaling raising new perspectives in the study of apical growth mechanisms. Signaling to other organelles and compartments is also likely to play key roles in the establishment of polarity.

We have discussed the hypothesis that, in nature, and upon a myriad of environmental (extracellular) cues, cellular responses involve flexible positioning of proteins, lipids and ion fluxes. Such dynamics may go partly unnoticed in the set-ups we devise for our experimental planning which are conditioned by the species under study, the technical approach, the pre-existing knowledge and, most importantly, by the requirements to test individual stimuli. Thus, transient gradients or peaks of activity/localization may or not be recorded (even dismissed) depending on the experimental set-up, cellular conditions and our biased previous background. Large single cell analysis and settings mimicking (or bearing in mind) the natural environment where cells develop, are required but may be difficult to implement. To compare and interpret results obtained with different experimental as well as plant systems will be a challenge, but one that must be tackled.

### Conflict of Interest Statement

The authors declare that the research was conducted in the absence of any commercial or financial relationships that could be construed as a potential conflict of interest.
